# Association between cytokines and two circulating micro-RNAs and development of premature ventricular contractions-induced cardiomyopathy

**DOI:** 10.22038/ijbms.2019.36362.8662

**Published:** 2019-10

**Authors:** Behshid Ghadrdoost, Nahid Aboutaleb, Mahin Nikougoftar Zarif, Mojdeh Nakhlestani, Majid Haghjoo, Shahram Sameie

**Affiliations:** 1 Physiology Department, School of Medicine, Iran University of Medical Sciences, Tehran, Iran; 2 Cardiac Electrophysiology Research Center, Rajaie Cardiovascular Medical and Research Center, Iran University of Medical Sciences, Tehran, Iran; 3 Physiology Research Center, Physiology Department, Faculty of Medicine, Iran University of Medical Sciences, Tehran, Iran; 4 Blood Transfusion Research Center, High Institute for Research and Education in Transfusion Medicine, Tehran, Iran

**Keywords:** Cardiomyopathiy, Cytokines, Inflammation, MicroRNAs, Peripheral blood-mononuclear cells

## Abstract

**Objective(s):**

Recent progress in understanding the pathogenesis of premature ventricular contraction (PVC)-induced cardiomyopathy (PIC) has suggested a key role for inflammation. The aim of this study was to evaluate the expression of messenger RNAs (mRNAs) and the protein production of interleukin-6 (IL-6), IL-10, tumor necrosis factor alpha (TNF-α) and interferon-γ (IFN-γ) and two circulating micro-RNAs related to inflammation and cardiovascular disease; miR-155 and miR-146.

**Materials and Methods:**

The study population was comprised 25 patients with PIC and 25 patients with normal left ventricular ejection fraction despite frequent PVCs. TNF-α, IL-6, IL10, and IFN-γ levels were evaluated in peripheral blood mononuclear cells (PBMCs) by flow cytometry and their mRNAs were assessed by real time PCR. We analyzed circulating levels of these cytokines by enzyme linked immunosorbent assay (ELISA). Two circulating micro-RNAs, miR-155 and miR-146a, were also investigated.

**Results:**

The flow cytometry findings showed that the median fluorescence intensity (MFI) of antibodies reacted with the IL-6 and TNF-α were higher in PIC group than the control group (*P*-value<0.001). In ELISA, the levels of IL-6 (*P*-value<0.001) and TNF-α (*P*-value <0.001) and in RT-PCR the relative expression levels of IL-6 (*P*-value<0.001) and TNF-α (*P*-value<0.001) were significantly higher in the PIC group. The relative expression levels of miR-155 and miR-146a were not significantly different between 2 groups (*P*-value>0.05).

**Conclusion:**

In our patients with PIC, there was an elevation in the expression levels of IL-6 and TNF-α in PBMCs. This finding may provide further insights into the inflammatory pathways involved in PIC.

## Introduction

Frequent premature ventricular contractions (PVCs) have been associated with nonischemic cardiomyopathy, referred to as PVC-induced cardiomyopathy (PIC) (1). Frequent PVCs that are defined as ventricular bigeminy with a coupling interval of 240 msec lead to ventricular dysfunction, although there are no detectable structural abnormalities (2). The prevalence of PIC is estimated as only 5% to 7% among patients with a PVC burden >10% (3).

The mechanism of PIC is not completely understood (3, 4). Significant controversy still exists regarding whether or how chronic PVCs may induce electrical remodeling, which may contribute to the increased risk of cardiomyopathy; nonetheless, bioactive molecules that are activated and released such as inflammatory cytokines can be considered as potential components in the development of PIC (5, 6).

Release of cytokines results in further activation of the adaptive immune system, including lymphocytes. Lymphocytes are the major cellular components of the adaptive immune response and are considered as the main components of peripheral blood mononuclear cells (PBMCs), which also include monocytes (7). PBMCs are important players in the inflammatory process, with their recruitment constituting one of the earliest events in cardiovascular events, and cytokines play a significant role in promoting the migration and activation of these cells at the site of inflammation (8).

Pro-inflammatory cytokines including tumor necrosis factor-α (TNF-α), interferon gamma (IFN-γ) and interleukin-6 (IL-6) appear to make a significant contribution to the pathophysiology of cardiomyopathy and heart failure (9). In contrast to pro-inflammatory mediators, regulatory pathways involving anti-inflammatory cytokines such as IL-10 influence the inflammatory activation of monocytes (8). 

Micro-RNAs are regulatory non-coding RNAs, which regulate protein coding gene transcripts’ expression, play an essential role in inflammation processes. MiR-146 and miR-155 are two most studied micro-RNAs, known for their multiple roles in the control of the immune processes and inflammatory disease as well as cardiovascular disease (10, 11)

Therefore, since we tried to find the role of inflammation in the development of PIC, we sought to investigate the association between increased inflammatory biomarkers including interlukin-6, interlukin-10, TNF-α and interferon-γ and two circulating micro-RNAs related to inflammation and the development of PIC.

## Materials and Methods

***Study design and population***

This case-control study was approved by the Institutional Review Board at Iran University of Medical Sciences and was performed according to the Helsinki Declaration of the World Medical Association (2000). Patient selection was conducted in our center between May 2016 and May 2017. 

The study population consisted of 50 patients with frequent PVCs who referred to the electrophysiology laboratory for radiofrequency ablation (RFA). A frequent PVC was defined as a greater than 20% of the total number of heart beats per 24 hr or a burden of 44% at baseline 24 hr Holter monitoring (12, 13). All the patients were refractory to at least 1 antiarrhythmic medication. Antiarrhythmic medications were discontinued 5 half-lives before the ablation procedure, except for amiodarone.

Patients with structural heart diseases including valvular heart disease and congenital heart disease as assessed with echocardiography or angiography were excluded from the study. The other exclusion criteria were any condition that causes left ventricular (LV) remodeling, including: history of chemotherapy with cardiotoxic drugs, coronary artery disease, systemic arterial hypertension, thyroid dysfunction, inflammatory and infectious disease, the use of anti-inflammatory drugs, alcoholism, and diabetes mellitus.

The case group consisted of 25 patients with PIC based on the echocardiographic parameters. (PIC was defined as a left ventricular ejection fraction (LVEF) <50% in the presence of frequent PVCs that was normalized or improved by ≥10% after RFA (14). 

The control group was comprised 25 patients with a normal LVEF and LV volume despite frequent PVCs.

*Echocardiography*

The echocardiographic studies were performed via the Simpson method using a Vivid 7 echocardiograph (General Electric, Milwaukee, WI, USA). The LVEF and LV dimensions were measured before ablation in all the patients. Post-extra-systolic augmentation was avoided by obtaining the measurements after at least 2 consecutive sinus beats. Further echocardiography was performed after ablation to determine whether the LVEF and LV dimensions were normalized.

***Isolation of PBMCs***

10 ml of peripheral blood was collected from each patient in both groups of case and control. Heparinized fresh whole blood was diluted 1:1 with phosphate-buffered saline (PBS) solution. Then, the PBMC fraction was separated by Ficoll-Hypaque (Sigma Aldrich, USA) centrifugation at 2200 revolutions per minute (RPM) for 25 minutes at 18 ^°^C. PBMCs were collected and washed twice with PBS and suspended in PBS (1:10). PBMCs were counted using a Scepter automatic cell counter (Merck Millipore).

***Detection of cytokine expression by flow cytometry***

For intracytoplasmic staining of target proteins, cells were fixed and permeabilized using unique perm kit (Invitrogen, USA) according to the manufacturer’s instruction. Then, 10 ul of monoclonal mouse Anti-human IFN-γ, TNF-α, IL-6 and polyclonal rabbit anti-human IL-10 (all from Abcam, USA) were added to 1×10^5^ PBMCs in separate test tubes. In parallel, mouse and rabbit antibodies were used as negative isotype controls. After 30 min incubation in 4 ^°^C, cells were washed using PBS and re-incubated with 10 ul of Fluorescein Isothiocyanate (FITC)-conjugated Rabbit anti- mouse and FITC-conjugated Goat anti-rabbit antibodies (all from BD Biosciences, USA) as secondary antibodies in 4 ^°^C for 30 min. After that, samples were analyzed by fluorescence-activated cell sorter (FACS) Caliber flow cytometer (Becton Dickinson, Franklin Lakes, NJ). Flow cytometry data was analyzed by FlowJo software.

***Enzyme-linked immunosorbent assay***

The plasma concentrations of cytokines in the two groups were measured with specific sandwich enzyme-linked immunosorbent assay (ELISA) method as described in manufacturer’s instruction. For ELISA analysis, following kits were used: IL-6 and TNF-α (Bender, eBioscience, Austria), IL10 and IFN-γ (IBL, Germany) 

***Real-time polymerase chain reaction***

Total RNA was isolated from whole blood using QIAzol (QIAGEN, Cat No. /ID: 79306) with a slight modification. Total RNA was quantified and partially qualified with an Ultrospec 3100 Pro spectrophotometer (Amersham Pharmacia Biotech, Piscataway, NJ). Additionally, agarose gel electrophoresis was performed for a better evaluation of the RNA quality.

cDNA was synthesized with a QuantiTect Reverse Transcription Kit (QIAGEN, Cat No./ID: 205313) according to the manufacturer’s protocol. 

Real-time polymerase chain reaction (RT- PCR) for cytokines was performed using a QuantiTect SYBR Green PCR Kit (QIAGEN, Cat No/ID: 204143) in accordance with the manufacturer’s protocol. Thermal profiling and data acquisition were performed via Corbett RT- PCR with its default parameter and thermal profile according to the manufacturer’s recommendation for the SYBR Green Kit. 

Furthermore, miR-155 and miR-146a and their internal control RNU48 were determined with a TaqMan Reverse Transcription Kit (Applied Biosystems, Foster City, Calif, USA) and via the TaqMan miRNA assay (Applied Biosystems) according to the manufacturer’s guidelines. The relative expression for a gene of interest in a sample was given by: 2–ΔΔCT.

***PCR primer design***

The SYBR Green primers of the genes of the cytokines were designed with the Primers Express 3.0 software. All the accession numbers and intron-exon positions were selected from NCBI Ensembl database. All the primers were with thermodynamically favorable parameters (Table1). Sequences of the primer for the two micro-RNAs are shown in Table 2.

***Statistical analysis***

The statistical analyses were performed with GraphPad Prism 5.01 (GraphPad Software, 2007, CA, USA). The data were expressed as means±standard errors of the mean (SEMs). The T- Student-test was used to calculate the significance of the difference between the groups and a *P* value of less than 0.05 was considered statistically significant. 

## Results

***Background data***

The demographic and echocardiographic data of patients in case and control group are shown in Table 3.

***Flow cytometry analysis of the intracellular cytokines***

**Figure 1:**
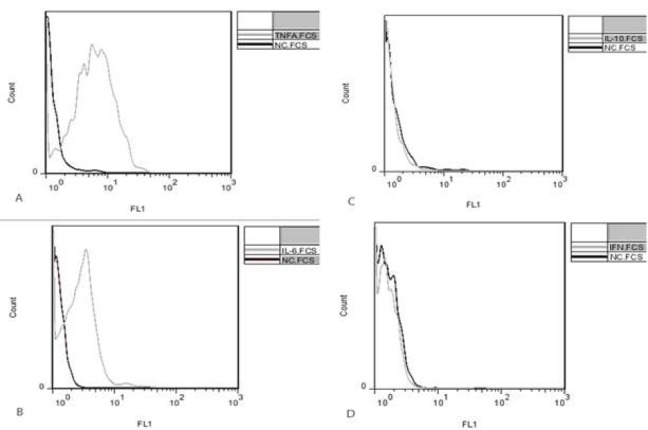
**Flow cytometry histograms represent intracellular IL-10, IL-6, TNF-α and IFN-γ expression in PBMC in case group. Clear and shaded histograms show antibody-reacted cytokines and negative controls, respectively. A shift to the right demonstrates an increase in TNF-α (A) and IL-6 (B) expression. There was no significant change in IL-10 (C) and IFN-γ (D) expression. IFNγ: Interferon gamma, IL-6: Interleukin 6, IL-10: Interleukin 10, TNF-α: Tumor necrosis factor alpha, peripheral blood mononuclear cells (PBMC)**

**Figure 2:**
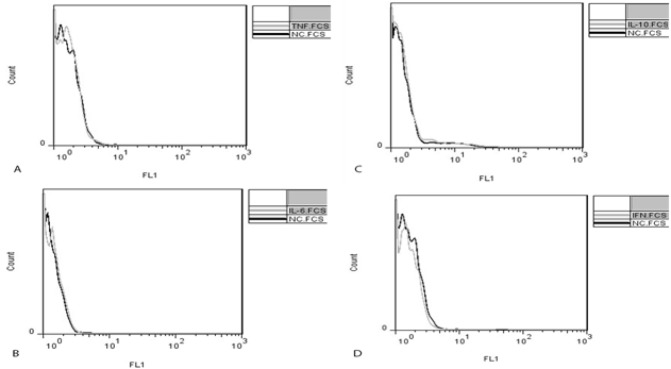
**Flow cytometry histograms represent intracellular IL-10, IL-6, TNF-α and IFN-γ expression in PBMC in control group. Clear and shaded histograms show antibody-reacted cytokines and negative controls, respectively. There was no significant change in TNF-α (A), IL-6 (B), lIL-10 (C) and IFN-γ (D) expression in control group. IFNγ: Interferon gamma, IL-6: Interleukin 6, IL-10: Interleukin 10, TNF-α: Tumor necrosis factor alpha, peripheral blood mononuclear cells (PBMC)**

To compare IL-10, IL-6, TNF-α and IFN-γ production by PBMCs in PIC and control groups, cytokine profiles were investigated in two groups. The PBMC population was first gated based on size (FS) and granularity (SSC) scattering of the cells in flow cytometry. The median fluorescence intensity (MFI) of reacted cells with specific conjugated antibodies was detected as median expression of target cytokines in cells (Figure 1, 2).

In patients with PIC (case group), a shift to the right demonstrates an increase in IL6 and TNF-α expression in cells (Figure 1a, 1b); however there were no significant increase in IL-10 and IFN-γ expression according to the results (Figure 1c, 1d).

In control group, there were no significant shifts in the histogram for any of the cytokines (Figure 2).

**Figure 3:**
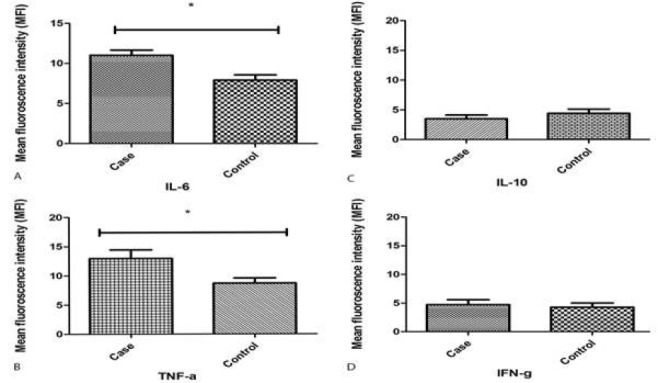
**Mean fluorescence intensity (MFI) of antibody reaction for TNF-α (A) and IL-6 (B). TNF-α and IL-6 expressions are significantly higher in patients with premature ventricular contraction (PVC)-induced cardiomyopathy compared to control group. There are no statistically significant differences in IL-10 (C) and IFN- γ (D) expression. TNF-α: Tumor necrosis factor alpha, IL-6: Interleukin 6, IL-10: Interleukin 10, IFNγ: Interferon gamma**

The MFI for each cytokine is shown in Figure 3, which clearly demonstrates that IL-6 and TNF-α expression are higher in patients with PIC in comparison with control group (*P*-value<0.001). No statistically significant differences were observed in the expression of IFN-γ (*P*-value: 0.32) and IL-10 (*P*-value: 0.07) between study groups. 

***Plasma levels of the cytokines***

**Figure 4:**
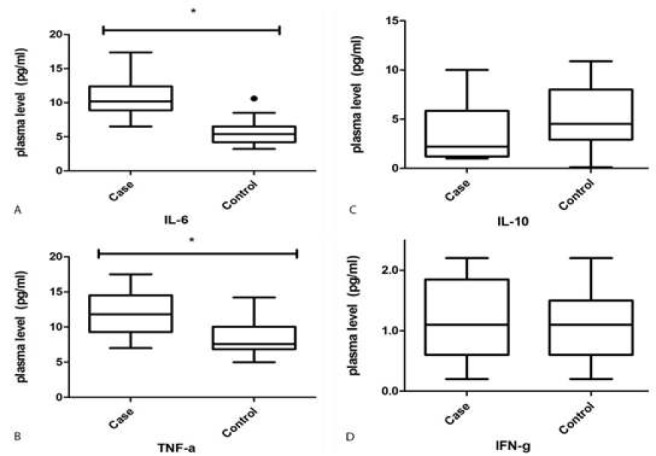
**In ELISA, the results of plasma levels of IL-10, IL-6, TNF-α and IFN-γ in patients with premature ventricular contraction (PVC)-induced cardiomyopathy compared to control group showed that: the levels of IL-6 (A) and TNF-α (B) were significantly higher in case group. There was no significant statistically difference in plasma level of IL-10 (C) and IFN-γ (D) between groups. IFNγ: Interferon gamma, IL-6: Interleukin 6, IL-10: Interleukin 10, TNF-α: Tumor necrosis factor alpha**

A comparison of the plasma levels of IL-10, IL-6, TNF-α and IFN-γ between the patients with PIC and the control group showed that the levels of IL-6 and TNF-α were significantly higher in the case group (IL-6= 10.76±0.54 pg/ml in the case group vs 5.61±0.36 pg/ml in the control group, *P*-value <0.001; TNF-α= 11.99±0.63 pg/ml in the case group vs 8.81±0.54 pg/ml in the control group, *P*-value: 0.0004); however, there were no statistically significant differences in the plasma levels of IFN-γ and IL-10 between the 2 groups (IFN-γ= 1.21±0.13 pg/ml in the case group vs 1.07±0.10 pg/ml in the control group, *P*-value: 0.41; IL-10= 3.61±0.58 pg/ml in the case group vs 5.18±0.59 pg/ml in the control group, *P*-value: 0.06) (Figure 4).

***Cytokines mRNA levels by RT-PCR***

**Figure 5:**
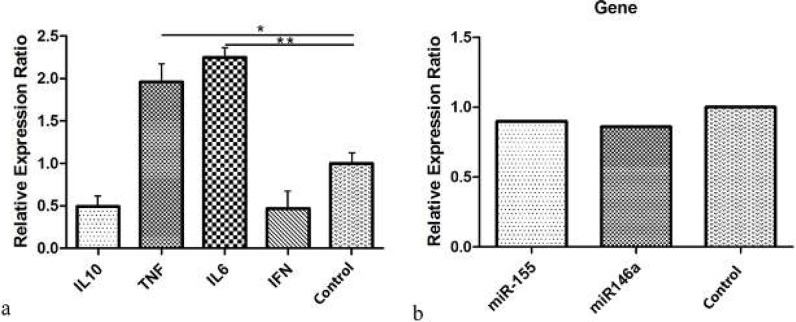
**a) Real-time PCR showed that the relative expression levels of IL-6 and TNF-α in patient with premature ventricular contraction (PVC)-induced cardiomyopathy were significantly increased compared to control. Glyceraldehyde 3-phosphate dehydrogenase (GADPH) was used as an internal control. b) The relative expression levels of miR-155 and miR-146a were not statistically significant in case compared to control. RNU48 was used as an internal control. IFNγ: Interferon gamma, IL-6: Interleukin 6, IL-10: Interleukin 10, TNF-α: Tumor necrosis factor alpha**

Based on the results of the quantitative RT- PCR, the relative expression levels of IL-6 and TNF-α were significantly increased in the patients with PIC in comparison with the controls (*P*-value<0.001), but there were no statistically significant differences in the relative expression levels of IFN-γ and IL-10 between the case and control groups (Figure 5A). 

***Levels of miR-155 and miR-146a by RT-PCR***

The relative expression levels of miR-155 and miR-146a according to the quantitative RT- PCR were not statistically different between the case and control groups (*P*-value: 0.5 and *P*-value: 0.9, respectively) (Figure 5B).

## Discussion

**The migration of leukocytes from the circulation to the areas of myocardial inflammation induced by electrical remodeling due to frequent PVCs and an increased expression of pro-inflammatory cytokines may contribute to an impaired cardiac function through cardiomyocyte apoptosis, inflammatory response, cardiac hypertrophy and matrix metalloproteinase activation (** 15 **); however, in this study we studied only inflammatory responses via releasing cytokines.**

**Cytokines produced by PBMCs may be a potential biochemical mediator of the progression of LV dysfunction via cellular and humoral immune pathways in PBMCs. Experimental models have suggested that activated PMBCs, such as cytotoxic T lymphocytes, may have a role in inducing and augmenting myocardial damage by secreting cytotoxic cytokines (** 16 **).**

**In our study, to evaluate the role of inflammatory cytokines and micro-RNAs in the etiology of PVC-induced cardiac dysfunction (PIC), we compared plasma levels of TNF-α, IL-6, IL-10 and IFN-γ by flow cytometry and ELISA, and mRNA by qPCR, and also abundance of miR-155 and miR-146a** **between patients with PIC and patients with PVCs but preserved cardiac function. Expression levels of TNF-α and IL-6 were elevated in PIC patients relative to those with normal cardiac function. There were no differences in abundance of the levels of other cytokines, or of the studied micro-RNAs.**

Our findings showed a high level of IL-6 mRNA and protein along with elevated levels of serum IL-6 in the patients with PIC. The expression of IL-6 is rapidly induced when cells like resting macrophage are exposed to proper stimuli such as IL-1 and TNF-α. Leukocytes are one of the non-cardiac sources of IL-6 in the peripheral circulation (as is evidenced by differences in the arterial-venous plasma concentration of IL-6), and a rise in their level can be deemed a marker of inflammation activation. Thus, a peripheral source may contribute to the development of inflammation in pathogenesis of PIC (9, 17).

*: ***P-*** **value <0.005**

*: ***P-*** **value <0.005**

*, **: ***P-*** **value<0.005**

**Table 1:** **Primer sequences of genes and PCR condition**

Gene	Length	T_m_ (^º^C)	Accession number
IL-6			
**F: TCAACCCCCAATAAATATAGGACTG**	25	**59**	**NM_001318095-1**
**R: TGTTACATGTTTGTGGAGAAGGAG**	24	**59**	
Product	133		
TNF-α			
**F: CCCAGGGACCTCTCTCTAATC**	21	**58.40**	**NM_000594.3**
**R: ATGGGCTACAGGCTTGTCACT**	21	**61.46**	
Product	84		
IL-10			
**F: CGAGATGCCTTCAGCAGAGT**	20	**60**	**NM_000572.2**
**R: GGCAACCCAGGTAACCCTTA**	20	**60**	
Product	109		
IFN-γ			
**F: CTGTTACTGCCAGGACCCAT**	20	**59.38**	**NM_000619.2**
**R: TCTGTCACTCTCCTCTTTCCA**	21	**57.76**	
Product	136		

IFNγ: Interferon gamma, IL-6: Interleukin 6, IL-10: Interleukin 10, TNF-α: Tumor necrosis factor alpha.

**Table 2:** **Primer sequences of miRNAs and PCR condition**

**miRNA**	**Sequence**	**(Length, Melt Temp)**
**has-miR-155-5p**	**>hsa-miR-155-5p MIMAT0000646**	
	**UUAAUGCUAAUCGUGAUAGGGGU**	**(23nt)**
**Stem-loop**	**GTCGTATCCAGTGCAGGGTCCGAGGTATTCGCACTGGATACGACACCCC**	**(49nt, 78’C)**
**Forward**	**CCAGCCGCTTAATGCTAATCGTGATA**	**(26nt, 66.5’C)**
**Universal Reverse**	**CCAGTGCAGGGTCCGAGGTA**	**(20nt, 67.2’C)**
**Amplicon**	**CCAGCCGCTTAATGCTAATCGTGATAGGGGTGTCGTATCCAGTGCGAATACCTCGGACCCTGCACTGG**	**(68nt, 79.5’C)**
**hsa-miR-146a-5p**	**> MIMAT0000449**	
	**UGAGAACUGAAUUCCAUGGGUU**	**(22nt)**
**Stem-loop**	**GTCGTATCGACTGCCAGGTCCGAGGTATTCGCAGTCGATACGACAACCC**	**(49nt, 77’C)**
**Forward**	**CCAGCCGTGAGAACTGAATTCCAT**	**(24nt, 66.5’C)**
**Specific Reverse**	**CGACTGCCAGGTCCGAGGTA**	**(20nt, 67.1’C)**
**Amplicon**	**CCAGCCGTGAGAACTGAATTCCATGGGTTGTCGTATCGACTGCGAATACCTCGGACCTGGCAGTCG**	**(66nt, 79.5’C)**
**RNU48**		
	**AGTGATGATGACCCCAGGTAACTCTGAGTGTGTCGCTGATGCCATCACCGCAGCGCTCTGACC**	**(63nt)**
**Stem-Loop**	**GTCGTATCCAGTGCAGGGTCCGAGGTATTCGCACTGGATACGACGGTCAG**	**(50nt, 71'C)**
**Forward**	**CTCTGAGTGTGTCGCTGATGCC**	**(22nt, 66.5'C)**
**Amplicon**	**CTCTGAGTGTGTCGCTGATGCCATCACCGCAGCGCTCTGACCGTCGTATCCAGTGCGAATACCTCGGACCCTGCACTGG**	**(79nt, 80.8'C)**

**Table 3:** **Demographic and echocardiographic data in case and control groups**

	**Case**	**Control**
**Age**	**47.33±12.32**	** 52.32±10.25**
**Sex (male)**	**13(52%)**	** 15(60%)**
**LVEF**	**30.20±5.11**	** 55.30±10.00**
**LV end-diastolic diameter**	**65.6±9.10**	**50.2±7.7**
**LV end-systolic diameter**	**49.5±4.5**	**37.4±4.7**

**LVEF: Left ventricular ejection fraction, LVEDD: Left ventricular end-diastolic diameter, LVESD: Left ventricular end-systolic diameter.**

In our study, TNF-α was another factor whose level increased both at the protein level and at the level of gene expression in PIC. Experimental studies have demonstrated that cardiac hypertrophy and dilated cardiomyopathy occur in transgenic mouse with a selective overexpression of TNF-α, indicating the strong and direct effect of TNF-α on cardiomyocytes via the induction of apoptosis, the depression of contractility, and the downregulation of sarcomeric proteins in cardiomyocytes (17-19). It seems that leukocytes subclasses and multiple inflammatory mediators in the progression of PIC have the same pattern with heart failure and dilated cardiomyopathy (20).

Monocytes and macrophages, when stimulated by other cytokines or inflammatory mediators, are the major source of TNF-α. In this situation, the elevated levels of circulating TNF-α may promote LV remodeling including myocyte hypertrophy, alterations in fetal gene expression, progressive myocyte loss through apoptosis, and alterations in the extracellular matrix leading to a progressive LV dilation and a gradual decline in the ventricular systolic function within hours. Although locally synthesized TNF-α in cardiomyopathy may be remarkable, the level of TNF-α correlates more with the functional class rather than the LVEF, indicating perhaps that the heart is not the only source of this cytokine (9, 21). 

In this study, we assessed 2 circulating micro-RNAs related to inflammation and cardiovascular disease; miR-155 and miR-146a. miRNAs are novel class of biomarkers or treatment targets, which alterations of their physiological expression patterns are associated with several human cardiovascular diseases. Circulating miRNAs along with expression of miRNAs within myocardium are novel mechanisms in the regulation of signaling pathways associated with myocardial infarction, heart failure, and hypertrophy (22)

The relative expression level of several micro-RNAs was analyzed in the PBMCs of patients with variety of cardiovascular disease including coronary artery disease, cardiomyopathies and heart failure. miR-155 is one of the important micro-RNAs that contributes to the prevention of atherosclerosis development and progression by posttranscriptional regulation of the inflammatory response via mitogen-activated protein kinase (MAPK) pathway (23). miR-155 that was found in atherosclerotic plaques was derived from macrophages and smooth muscle cells, and it also regulates lipid uptake pathways in macrophages. Inhibition of miR-155 was found to increase both lipid uptake and inflammation in macrophages (24).

MiR-146a is a well-known miRNA associated with inflammatory autoimmune diseases, but it has prominent role in regulation of some pathway related to cardiovascular disease such as peripartum cardiomyopathy or coronary heart disease. Plasma miR-146a levels correlated with the severity of coronary atherosclerosis particularly among patients with elevated thyroid-stimulating hormone (TSH) levels (25).

These studies suggest that miR-155 and miR-146a play a crucial role in some cardiovascular diseases, which have the pathophysiological relevance with inflammation such as heart failure.

To understand the role of theses miRNAs in PIC, we performed RT- PCR and found that the expression of miR-155 and miR-146a was not increased significantly in the patients with PIC compared to the control group. 

MiR-146 and miR-155 are known as a regulator of inflammation in several diseases such as osteoarthritis and in some cardiovascular disease, which have the inflammatory pattern like heart failure (26-29). The expressions of these micro-RNAs are up-regulated by inflammatory factors such as IL-1 and TNF-α (30, 31). 

Nevertheless, we found no increase in the expression of these micro-RNAs in our patients with PIC. Therefore, more specific studies of the underlying mechanisms of the involvement of miR-146 and miR-155 in inflammatory cardiovascular diseases are required.

## Conclusion

This work demonstrates that the PBMCs of patients with PIC are able to produce high concentrations of several pro-inflammatory factors such as IL-6 and TNF-α. In this inflammatory situation, peripheral mononuclear cells are activated and they produce significant amounts of inflammatory mediators, which may contribute to the development of PIC. Further studies are needed to assess the role of micro-RNAs in various physiological processes of PIC development. The exact signaling pathways and molecular mechanisms involved, however, will require further investigations. 
